# The use of whole genome amplification to study chromosomal changes in prostate cancer: insights into genome-wide signature of preneoplasia associated with cancer progression

**DOI:** 10.1186/1471-2164-7-65

**Published:** 2006-03-30

**Authors:** Simon Hughes, Maisa Yoshimoto, Ben Beheshti, Richard S Houlston, Jeremy A Squire, Andrew Evans

**Affiliations:** 1 Applied Molecular Oncology, Ontario Cancer Institute, Princess Margaret Hospital, Toronto, Ontario M5G 2M9, Canada; 2 Faculty of Medicine, University of Toronto, Toronto, Ontario M5G 2M9, Canada; 3 Section of Cancer Genetics, Institute of Cancer Research, Sutton SM2 5NG, UK; 4 Department of Medical Biophysics, Faculty of Medicine, University of Toronto, Toronto, Ontario M5G 2M9, Canada; 5 Department of Pathology, Princess Margaret Hospital, Toronto, Ontario M5G 2M9, Canada; 6 Department of Laboratory Medicine and Pathobiology, University of Toronto, Toronto, Ontario M5G 1L5, Canada

## Abstract

**Background:**

Prostate cancer (CaP) is a disease with multifactorial etiology that includes both genetic and environmental components. The knowledge of the genetic basis of CaP has increased over the past years, mainly in the pathways that underlie tumourigenesis, progression and drug resistance. The vast majority of cases of CaP are adenocarcinomas that likely develop through a pre-malignant lesion and high-grade prostatic intraepithelial neoplasia (HPIN). Histologically, CaP is a heterogeneous disease consisting of multiple, discrete foci of invasive carcinoma and HPIN that are commonly interspersed with benign glands and stroma. This admixture with benign tissue can complicate genomic analyses in CaP. Specifically, when DNA is bulk-extracted the genetic information obtained represents an average for all of the cells within the sample.

**Results:**

To minimize this problem, we obtained DNA from individual foci of HPIN and CaP by laser capture microdissection (LCM). The small quantities of DNA thus obtained were then amplified by means of multiple-displacement amplification (MDA), for use in genomic DNA array comparative genomic hybridisation (gaCGH). Recurrent chromosome copy number abnormalities (CNAs) were observed in both HPIN and CaP. In HPIN, chromosomal imbalances involving chromosome 8 where common, whilst in CaP additional chromosomal changes involving chromosomes 6, 10, 13 and 16 where also frequently observed.

**Conclusion:**

An overall increase in chromosomal changes was seen in CaP compared to HPIN, suggesting a universal breakdown in chromosomal stability. The accumulation of CNAs, which occurs during this process is non-random and may indicate chromosomal regions important in tumourigenesis. It is therefore likely that the alterations in copy number are part of a programmed cycle of events that promote tumour development, progression and survival. The combination of LCM, MDA and gaCGH is ideally suited for the identification of CNAs from small cell clusters and may assist in the discovery of potential genomic markers for early diagnosis, or identify the location of tumour suppressor genes (TSG) or oncogenes previously unreported in HPIN and CaP.

## Background

The worldwide occurrence CaP is increasing and it has now overtaken lung cancer as the most commonly diagnosed malignancy in men in the Western World. In spite of significant progress in its clinical management, comparatively little is known about the aetiology of the disease and predicting outcome is still a challenge for most clinicians. Histopathologically, CaP displays considerable heterogeneity and can contain a substantial admixture of pre-malignant HPIN glands within cancerous foci. HPIN is currently considered to be the most likely precursor to invasive CaP [1]. However, most of these foci are latent and rarely develop into clinically detectable cancer. Unfortunately, a significant number (3%) do progress and can become aggressive and lethal [2]. The major difficulty facing clinicians is the identification of patients presenting with early stage CaP, who are likely to develop life-threatening disease. As a result, intensive research is currently underway to identify the key alterations that may prove to be important for both classification and prognosis prediction.

The advent and development of CGH, which started in the early 1990's [3], has revolutionised cytogenetics and allowed for genome-wide screening of numerous cancer types and the identification of genomic CNA, which may contribute to cancer development and progression. The accumulation of genetic changes that occurs during the stepwise evolution from normal tissue to metastasis, although likely due to increased genetic instability, may indicate the chromosomal locations of TSG or oncogenes that are important in tumourigenesis. When first introduced, CGH used metaphase chromosomes targets to identify CNAs. However, recent advances have substituted these with arrayed DNA sequences that provide higher resolution, 1 Mb versus 10 Mb, and greater ease of analysis.

Whole genome scans of CaP patients and cell lines have identified consistent, chromosomal alterations, which include recurrent loss of chromosomal regions from 5p, 6q, 8p, 10q, 13, 16q and 17p, in addition to gain on 1q, 3q 7p, 7q, 8q 11p, 17q and Xpq [4-6]. Nonetheless, one potential limitation with current CGH methodology is its requirement for microgram quantities of genomic DNA. When studying cell lines this is not necessarily a matter for concern, as it is relatively straightforward to produce a sufficient quantity of clonal cells to obtain many micrograms of DNA. However, when studying patient tissue if DNA is "bulk extracted" from a heterogeneous non-clonal tumour mass the data obtained will represent an average value for all cells within the specimen and any clinically informative genetic changes, restricted to small cell clusters, may be masked. As a result, when studying a heterogeneous tissue, such as prostate, it is essential to obtain pure cell populations via methods such as macrodissection or LCM. The DNA extracted from the dissected material can then be amplified using a whole genome amplification (WGA) technique [7], such as MDA [8-11]. The combination of LCM and WGA can generate sufficient amounts of DNA for extensive genome analyses. Several large studies have demonstrated the ability of MDA to accurately amplify human genomic DNA. When used in SNP genotyping [12,13] an estimated 99.82% of the genome is covered with a reported concordance, to non-amplified DNA, of greater than 99% [14]. In addition, the sequence error rate is only 7.6 × 10^6 ^[13] and previous results obtained from gaCGH, using amplified DNA, are almost indistinguishable from those obtained from non-amplified DNA [10,15].

An understanding of the molecular mechanisms behind cancer development will enable identification of molecular and cytogenetic biomarker(s) that may be useful in predicting early transformation into an aggressive phenotype, as well as, providing fundamental insights into the regulatory pathways of genome  integrity that may lead to multistep field cancerization and HPIN and CaP. In the present study, we describe the use of LCM, MDA and gaCGH, using a 1 Mb array (Spectral Genomics, USA) to investigate copy number changes occurring in prostate cancer by the analysis of 7 prostatic samples containing HPIN and 8 CaP specimens.

## Results

To determine whether whole genomic amplification exhibited bias or a distortion of imbalance assignments, control CGH experiments comparing the results obtained before and after amplification (normal male versus normal male, normal male versus normal female and DNA from a cancer cell line versus normal control DNA) from the same original DNA samples were performed. These are presented in Additional Files 1, 2, 3 and it is evident that no major distortion of imbalance was introduced by amplification of genomic DNA.

**Figure 1:**
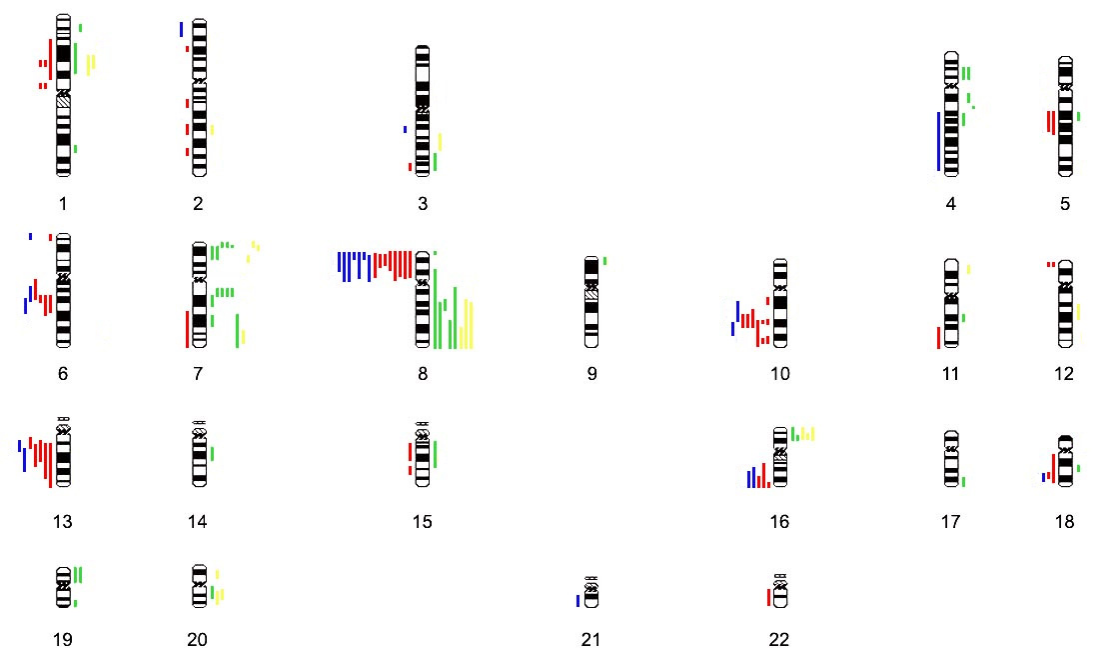
Chromosomal alterations observed for HPIN and CaP DNA samples. Blue bars indicate loss in HPIN and yellow bars indicate gain in HPIN. Red bars indicate loss in CaP and green bars indicate gain in CaP.

**Figure 2:**
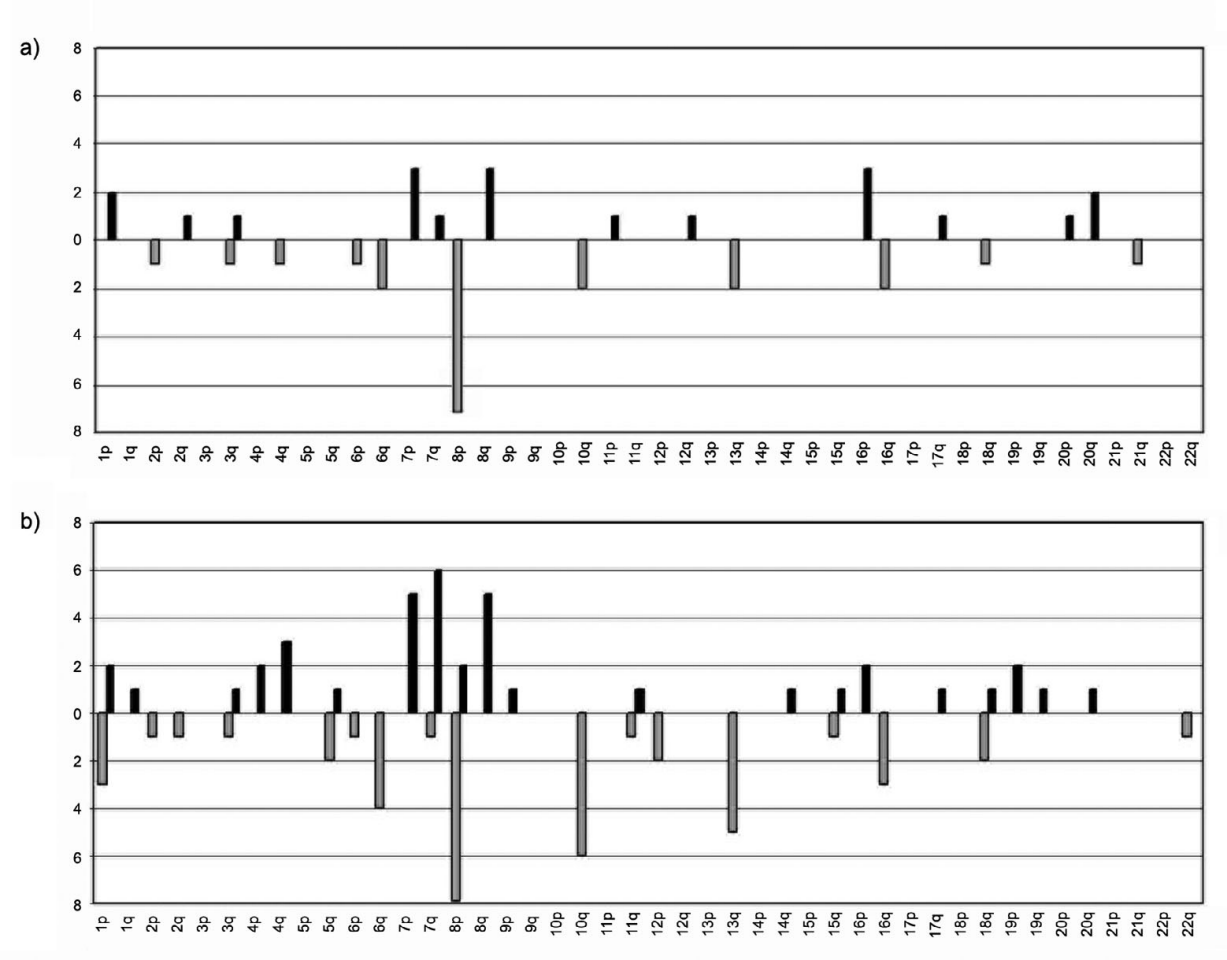
Summary of chromosomal losses and gains in a) HPIN (n = 7) and b) CaP (n = 8). Number of samples of each type with gain or loss of the chromosome arm is shown by black and grey bars, respectively.

A summary of the chromosomal CNAs detected by CGH for the 7 HPIN and 8 CaP samples is shown in Table 1 and 2 and displayed graphically in Figure 1 (for HPIN blue = loss, yellow = gain; for CaP red = loss, green = gain). Figure 2 shows the frequency of CNAs detected at the level of each chromosomes arm for HPIN and CaP. The general pattern of loss or gain was very similar in the HPIN and CaP samples, however CaP samples possessed significantly more aberrations (90 CNAs) than HPIN samples (41 CNAs) (P<0.001, chi square test). The average number of CNAs for the 7 HPIN samples was 5.8 (range 3 to 8, median 6). For the 8 CaP samples an average of 11.25 (range 5 to 18, median 11.5) amplifications or deletions were observed. When studied at the 850 cytoband level (minus X and Y chromosome cytobands), HPIN samples had the total of 65 affected bands (average 9, range 4 to 14) and CaP samples 143 affected breakpoints bands (average 18, range 8 to 27). This difference is statistically significant (P<0.001, chi square test). In addition, for both the HPIN and CaP samples there were a significantly greater number of cytobands lost than gained (P<0.01, chi squared test) (Table 1).

In the 7 HPIN samples recurrent chromosomal changes, detected in greater  than 25% of cases, were losses found on 8p (7 out of 7 cases, 100%), 6q,  13q, 16q (2 out of 7 cases, 28.5%, each), whereas gains were found on 8q  and 16p (3 out of 7 cases, 43%, each), 1p, 7p and 20q (2 out of 7 cases,  28.5%, each). For the 8 CaP samples recurrent chromosomal changes,  detected in greater than 25% of tumours, were losses on 8p (8 out of 8  cases, 100%), 10q (6 out of 8 cases, 75%), 13q (5 out of 8 cases,  62.5%), 6q (4 out of 8 cases, 50%), 1p, 16q (3 our of 8 cases, 37.5%),  5q, 12p, 18q (2 out of 8 cases, 25%, each) and gains on 7q (6 out of 8  cases, 75%), 7p, 8q (5 out of 8 cases, 62.5%, each), 4p, 16p, 19p (2 out  of 8 cases, 25%).  

Small consensus regions of consistent CNA were observed for both HPIN and CaP samples (Table 3), in >25% of samples. For example, 8p region was consistently lost in HPIN (100%), but within the cytoband 8p11.23-p23.2 that was commonly lost in >50% of the HPIN samples. Similarly, 8p was consistently lost in CaP (100%), but within the cytoband 8p11.22-p23.2 that was commonly lost in >60% of the CaP samples. A list of the genes present within these consensus or commonly lost regions is displayed in Table 3.

## Discussion

Chromosome copy number abnormalities (CNAs) are common in most cancers, with specific regions of amplification or deletion being associated with specific tumour types, stages or outcomes [16-18]. The introduction of metaphase CGH [3] to study these CNAs has eliminated the requirement to obtain metaphase spreads from the tumour samples, which was often challenging due to technical difficulties in culturing certain tissues. Though metaphase CGH allows for more samples to be examined, it provides a relatively limited resolution and still requires substantial cytogenetic experience to analyse the results. The recent development of array CGH has opened up the field of CGH research and permitted more laboratories to study an ever-increasing number of tumour types and stages. The application of BAC microarrays to analyse human samples is straightforward and their high-throughput nature makes them the method of choice for rapid detection of genetic alterations.

When analysing heterogeneous tumour samples, the gaCGH results obtained from "bulk extracted" DNA are likely to be inaccurate. However, with the use of LCM and MDA we have been to able obtain highly purified HPIN and CaP DNA and thus identify the particular chromosomal changes associated with the two disease stages.

In this study, we have used a 2,400-element BAC microarray with a resolution of ~1 Mb to study CNAs in a set of 15 patient samples comprised of 7 HPIN cases and 8 CaP cases. For the 7 HPIN cases, 41 genomic alterations (20 gains, 21 losses) were identified, in contrast to the 90 genomic alterations (38 gains, 52 losses) seen for the 8 CaP cases. As with other cancers, CaP development and progression is likely to be the outcome of a series of stepwise genetic changes. The accumulation of CNAs, which occurs during this process, although likely due to increased genetic instability, is non-random and may indicate chromosomal regions important in tumourigenesis. It is suggested that failure in the fidelity of homologous recombination within the repetitive sequences, that comprise the kinetochore complex, could lead to recurrent loss of 8p and gain of 8q by rearrangement of chromosome 8-specific alphoid centromeric sequences. Thus, the high fidelity process of homologous recombination can be the major DNA repair pathway, which is indispensable for the maintenance of genetic stability.

Examination of our array results indicates that aberrations involving parts or all of 1p, 6q, 7p, 7q, 8p, 8q, 10q, 13q 16p and 16q are most common, which is concordant with previous metaphase [19,20] and array CGH [4,21] results. It is therefore likely that the alterations in copy number are part of a programmed cycle of events that promote tumour development and progression as well having an impact on disease-specific survival.

A comparison of the CNAs present in the HPIN and CaP samples identified a significant increase in copy number for 7q (P<0.01, chi square test) and a significantly increased frequency of loss for 10q (P<0.01, chi square test) and 13q (P<0.0001, chi square test). In genotype/phenotype correlations, gain of chromosome 7q [22] and loss of 13q [23] have been associated with advancing tumour stage and aggressiveness, which is in agreement with the results presented here. However, gain of 8q [24] and loss of 16q [25] have also been linked to tumour progression, but our data do not show any significant difference for these CNAs in our HPIN and CaP samples. This would suggest that 8q and 16q CNA's are likely to be early events in tumourigenesis. In addition, they may also identify HPIN and CaP samples that are likely to progress.

Apart from the commonly reported CNAs, additional alterations that have been less frequently reported in earlier CGH studies have also been identified. These include gains on 12q (HPIN) and loss of 4q and 10q (HPIN and CaP). Whether the identification of 12q and 4q regions will provide additional insight into CaP progression is not yet clear. Further analysis using a platform such as tissue microarrays, which permits the screening of different disease stages from large patient cohorts, will help better identify their frequency and also the potential use of genomic imbalance in diagnosis of CaP. The value of genomic analysis in CaP was recently demonstrated by the discovery of a high frequency of chromosomal translocations leading to rearrangement of the fusion oncoproteins *ERG *or *ETV1 *with *TMPRSS2 *[26].

A common problem when analysing archival tissue is the availability of a suitable quality and quantity of RNA to study expression changes. Though RNA amplification techniques [27] can generate a sufficient quantity, obtaining RNA of the required quality is often challenging. Previous reports have demonstrated a relationship between alterations in chromosomal copy number and alterations in gene expression [28,29]. The ability to distinguish these regions will point to genes, which may either directly (tumour suppressor gene loss or oncogene gain) or indirectly contribute to tumour development and progression. As a result, CGH can be used as a surrogate for gene identification. Candidate genes, which have previously been implicated in CaP have been highlighted in bold in Table 3. The roles of *MYC *[30-32], *PSCA *[33-35], and *MDM2 *[36-38] have all been well reported and alterations in gene dosage correlate well with their change in expression. However, other candidate genes have been less well studied. For example, *EBAG9*, whose increased expression in CaP is a negative prognostic indicator, has a potential role in progression by enabling cancer cells to evade the immune response [39]. *NBS1*, which has been identified as a founder mutation causing an increased susceptibility to prostate cancer [40], is involved in processing/repair of DNA double strand breaks and in cell cycle checkpoints, thus its deregulation will likely contribute to chromosomal instability. *FKHR*, which is a member of the *FOXO *forkhead transcription factor family, is thought to play a regulatory role in several cellular functions including cell proliferation and survival [41]. Loss of *FKHR *expression, as observed in CaP cell lines, is likely to abrogate this control leading to tumour cell growth. Though these genes have previously been implicated in CaP there are additional genes, including both oncogenes and tumour suppressor genes, which reside within all of the affected regions that may play an important role in the aetiology of the disease. Further analysis of these other candidates may identify their potential as molecular targets for diagnosis and treatment.

Several of the genes present within altered regions of the genome are associated with genetic pathways, indicating that these pathways are likely to be important in prostate tumourigenesis.

## Conclusion

The combination of techniques used in this study has allowed for the identification of consistent regions of copy number change, ranging from specific cytobands to whole chromosomes, starting with as little as 5–10 ng of DNA. Through our use of LCM, we have also identified several lower frequency CNAs which may not have been detected when using bulk extracted DNA due to the heterogeneous nature of prostate tissue. In addition, several interesting candidate genes have been observed within the altered chromosomal regions. Although some have already been extensively studied others, which have not, may prove to be of clinical importance. Genetic screening strategies that combine FISH and immunohistochemistry for the detection of various combinations of chromosomal gains and/or losses and altered gene expression are likely to be of great use in diagnosis and prognosis prediction.

## Methods

### Tissue accrual

Fresh prostate tissue was obtained from radical prostatectomies performed at The University Health Network (UHN), Toronto, with the informed patient consent and approval of the institutional research ethics board. The tissue was embedded in OCT frozen section medium (Stephens Scientific, Riverdale NJ, USA) and stored at -80°C until required. For all samples, the presence of HPIN and CaP was verified by histologic assessment of frozen sections by a urological pathologist (A.E.).

### Cohort selection

Patient samples for gaCGH analysis were selected based on two criteria; first, the presence of clearly identifiable regions of HPIN and/or CaP and second, the availability of good quality high molecular weight DNA. DNA quality was assessed by DNA extraction from a single tissue section and visualisation by agarose gel electrophoresis. A cohort consisting of 15 cases (7 HPIN and 8 CaP derived from different patients) were selected based on these criteria.

### Laser capture microdissection

The selected fresh-frozen prostate tissue samples were cut onto microscope slides (8 μm thickness) and stained using the HistoGene LCM frozen section staining kit (Arcturus, USA). Foci of HPIN and CaP were identified from stained prostate tissue sections and a minimum of 1000 cells were removed from these regions by LCM using the Cell Robotics LaserScissors system (Cell Robotics Inc., USA).

### DNA extraction

DNA was extracted using the QIAamp DNA Micro Kit (Qiagen, USA) and the DNA concentration was determined using the PicoGreen dsDNA Quantitation kit (Molecular Probes Inc., USA). Both procedures were performed following manufacturers instructions.

### Strand displacement amplification

DNA was amplified using the GenomiPhi Amplification Kit (Amersham Biosciences, USA) according to manufacturer's instructions. Briefly, amplification was carried out in two individual steps. The step 1 reaction mixture contained 5–10 ng (1000 to 2000 cell equivalents) of DNA (from diluted reference or test DNA) in 1 μl of sterile water and 9 μl of Sample Buffer. This mixture was heated at 95°C for 3 minutes and then chilled on ice. The step 2 reaction (amplification) mixture contained 9 μl of Reaction Buffer, 1 μl of Enzyme Mix and the 10 μl from Step 1. The amplification reaction was incubated at 30°C for 16–18 hours. The enzyme was inactivated by heating at 65°C for 10 minutes, followed by cooling to 4°C. This method of WGA consistently produced in excess of 5 μg of DNA from a starting concentration of 5–10 ng.

### Assessment of DNA quality and strand displacement amplification

Five microlitres of each amplification reaction was electrophoresed through a 1% agarose gel and stained with ethidium bromide in order to assess product yield and product length. All amplification products were purified by phenol-chloroform extraction and DNA concentration and purity were determined by measuring absorbance at A260 and A280.

### Spectral genomic BAC arrays

The genomic DNA arrays used in these experiments were obtained from Spectral Genomics Inc. and consist of approximately 2400 unique BAC and PAC clones, which provide an average genomic resolution of 1 Mb. CGH experiments were performed using the amplified patient DNA as the "test" and amplified female placental DNA as the "normal". In addition, control experiments were performed, corresponding to DNA before and after amplification (normal non-neoplastic prostate epithelial DNA versus normal non-neoplastic prostate epithelial DNA, normal non-neoplastic prostate epithelial DNA versus normal female placental DNA and DNA from a cancer cell line versus normal control DNA).

Reference DNA and test DNA were first digested overnight at 37°C using 10 units of *RsaI *(Invitrogen) in a 10 μl reaction. The digested DNA was labeled using the protocol optimized by Spectral Genomics, with separate labeling reactions for Cy3 and Cy5 being set up for both reference and test DNA. Briefly, labeling reaction were set up containing 2 μg of DNA, 20 μl of 2.5× random primer/reaction buffer mix (Invitrogen) and sterile water up to a final volume of 45 μl. The reaction mix was boiled for 5 minutes prior to cooling on ice and the addition of 2.5 μl of Spectral labeling buffer (Spectral Genomics, Houston, U.S.A), 1.5 μl of either Cy3-dCTP (1mM, Applied Biosystems, Foster City, U.S.A) or Cy5-dCTP (1 mM, Applied Biosystems) and 1 μl of Klenow Fragment (BioPrime labeling kit, Invitrogen). The reaction was mixed gently and then incubated for 2 hours at 37°C. Following incubation the reaction was stopped by the addition of 5 μl 0.5 M EDTA (pH8) and heating at 72°C for 10 minutes.

The Cy3 labeled test DNA was combined with the Cy5 labeled normal reference DNA and vice versa. Each combined probe was mixed with 45 μl of Spectral Hybridisation Buffer (Spectral Genomics), 11.3 μl of 5 M NaCl and 110 μl of room temperature isopropanol. The samples were incubated in the dark at room temperature for 10–15 minutes and centrifuged at 16,000 x g for 10 minutes and the supernatant discarded. The pellets were then washed with 500 μl of 70% ethanol. The supernatant was carefully removed and the pellets air-dried at room temperature in the dark. For hybridisation, the pellets were first resuspended in 10 μl of sterile water prior to being mixed with 30 μl of Spectral Hybridisation Buffer II (Spectral Genomics) by pipetting. The reconstituted probes were then incubated at 72°C for 10 minutes, placed on ice for 5 minutes and then incubate for 30 minutes at 37°C. The probes were hybridised to BAC arrays, covered with a 22 × 60 mm coverslip and incubated for 12–16 hours at 37°C in a humidified chamber.

The wash buffers, with the exception of Wash I, were pre-warmed to 50°C. The slides were gently dipped into and out of Wash I (2× SSC, 0.5% SDS) until the coverslip detached from the slide. The slides were then washed once in Wash II (2 × SSC, 50% deionized Formamide, pH 7.5) for 20 minutes, followed by successive washes in Wash III (2× SSC, 0.1% NP-40, pH 7.5) for 20 minutes and Wash IV (0.2× SSC, pH 7.5) for 10 minutes. All washes were performed at 50°C, with the exception of Wash I. The slides were briefly submerged in distilled deionized water for 5–10 seconds and centrifuged for 5 minutes at 750 rpm to dry.

### Data collection and analysis

The slides were scanned using an Axon GenePix 4000 A confocal scanner, each fluorescence signal was collected separately and quantified with the GenePix Pro 3.0 software (Axon Instruments, U.S.A). The data was normalised and analysed using Normalise Suite v2.4 [42], all regions of loss or gain were determined as those that were 2 standard deviations above the mean baseline for each separate sample.

## Authors' contributions

SH and MY designed and carried out the study, in addition to preparing the final manuscript for publication. BB helped with the analysis of the data. AE helped in the selection of cases and worked closely with JS in the study design and coordination.

## Supplementary Material

Additional File 1 Genomic comparison of gaCGH results obtained from non-amplified male DNA versus non-amplified male DNA hybridisation and amplified male DNA versus amplified male DNA hybridisation. Blue line indicates the chromosome position plotting of amplified DNA data. Black line indicates the chromosome position plotting of non-amplified DNA data. Click here for file

Additional File 2 Genomic comparison of gaCGH results obtained from non-amplified male versus non-amplified female DNA hybridisation and amplified male DNA versus female DNA hybridisation. Blue line indicates the chromosome position plotting of amplified DNA data. Black line indicates the chromosome position plotting of non-amplified DNA data. Click here for file

Additional File 3 Genomic comparison of gaCGH results obtained from non-amplified DNA from the colorectal cell line DLD1 versus non-amplified control DNA male hybridisation and amplified DNA from the colorectal cell line DLD1 versus amplified control DNA hybridisation. Blue line indicates the chromosome position plotting of amplified DNA data. Black line indicates the chromosome position plotting of non-amplified DNA data. Detection of gain and loss is shown by cyan and pink bars, corresponding to amplified and non-amplified DNA, respectively. Click here for file

Additional File 4 H&E sections show an example of LCM. Dissection of HPIN. **Top **picture represents the tissue before dissection, **middle **picture is after dissection, and **bottom **picture is the cap tissue. Click here for file

## Figures and Tables

**Table 1:** Frequency of chromosome copy number abnormalities and affected cytoband breakpoints in CaP and HPIN.

**Type**	**Breakpoints**			**CNAs**		
		
	**Loss**	**Gain**	**Total**	**Loss**	**Gain**	**Total**
CaP	84	59	143	52	38	90
HPIN	32	33	65	21	20	41
CaP+HPIN	116	92	208	73	58	131

**Table 2:** Chromosomal changes in microdissected HPIN and CaP DNA samples.

**Tumour**	**Type**	**Loss**	Gain
CaP 2-02	HPIN	3q13.33-q21.1; 6p24.1-pter; 8p11.1-p23.2; 13q14.3-q31.2	1p21.3-p31.1; 2q24.3-q31.1; 8q23.3-qter; 11p14.3-p15.1
CaP 5-02	HPIN	6q14.1-q16.3; 8p23.1-pter; 16q21-qter	1p22.3-p31.1; 7p15.1-p15.3; 8q13.3-qter
CaP 24-01	HPIN	2p23.1-p25.2; 4q22.1-q34.1; 8p11.23-pter	3q21.2-q24; 12q14.3-q21.33
CaP 37-02	HPIN	8p23.1-pter;	7p22.1-pter; 20q11.22-q13.11
CaP 70-01	HPIN	6q16.3-q22.1; 8p11.21-pter; 13q13.1-q14.3	7p21.3-p22.1; 8q21.11-qter; 16p12.3-pter; 20q11.23-q13.2
CaP 74-01	HPIN	8p21.1-pter; 10q21.3-q23.1; 18q21.32-q22.2; 21q21.3-qter	7q32.3-q36.1; 16p12.3-p13.13; 20p11.23-p12.1
CaP 36-02	HPIN	8p11.23-pter; 10q23.31-q25.1; 16q22.1-qter	16p12.2-pter; 17q24.2-qter
CaP 7-99	Cancer	2q14.2-q21.2; 5q14.1-q22.1; 6q16.1-q21; 8p21.3-p23.1; 10q22.1-q23.33; 13q14.11-q22.2	4q21.21-q21.23; 7p15.3-p21.3; 7q11.23
CaP 7-01	Cancer	1p21.1-p32.3; 2q33.1-q33.3; 5q14.1-q21.3; 6q16.1-q22.1; 8p11.23-pter; 10q23.31-qter; 13q12.13-q14.11	4p13-p15.1; 4q22.2-q24; 7q31.1-qter; 8q21.11-qter; 9p22.3-p24.1; 14q13.1-q21.3
CaP 7-02	Cancer	2q24.2-q31.1; 7q22.3-qter; 8p11.22-pter; 10q22.2-q23.33; 18q11.1-q22.3	
CaP 11-01	Cancer	8p11.21-pter; 10q22.2-q23.33; 12p13.2; 13q13.3-q32.2; 16q24.1-qter; 22q11.21-qter	1q32.1; 5q14.2-q14.3; 8q13.3-q21.2
CaP 11-02	Cancer	1p13.1-p13.2; 1p22.3; 2p16.3; 6q12-q16.1; 8p21.1-pter; 10q25.3-q26.11	7p22.1-pter; 7q11.23; 8q22.3-qter; 16p12.2-pter; 19p; 19q13.33-qter
CaP 14-00	Cancer	6p24.1-pter; 8p11.21-pter; 10q23.31-q23.32; 15q22.33-q24.1	1p21.3-p32.1; 3q25.33-q27.3; 4p13-p15.1; 4q13.1-q13.3; 7p15.3-p22.1; 7q21.11-q21.3; 7q31.1-q31.33; 8q11.22-qter; 11q14.1-14.3
CaP 14-02	Cancer	1p13.1-p13.2; 1p22.3; 6q16.1-q16.3; 8p21.2-p23.1; 8q12-qter; 10q21.1-q21.2; 10q23.1-q23.32; 10q25.2-q26.11; 11q22.3-qter; 12p13.2; 13q12.3-q21.33; 15q13.1-q21.3; 16q12.2-qter	7p22.1-pter; 7q11.23; 8p23.2-pter; 18q21.1; 20q11.21-q13.12
CaP 32-02	Cancer	3q26.33-q27.3; 8p11.22-p23.2; 13q13.3-qter; 16q23.1-qter; 18q21.33-q22.1	1p35.2-p36.11; 7p22.1-p22.2; 7q11.23; 15q11.2-q22.32; 16p12.2-13.11; 19p13.11-pter

**Table 3:** Consensus regions of copy number gain. Names in bold indicate those genes that have been implicated in prostate cancer. The * symbol indicates the same candidate genes from HPIN.

Chromosome	Region	Frequency	Candidate genes
*HPIN Gain*			
1p	1p22.3 – p31.1	37.5%	*IGFBP10*
2q	2q24.3 – q31.1	25%	*TLK1, ITGA6*
7p	7p22.1	37.5%	*NUDT1, **PDGFA***
	7p15.1 – p15.3	25%	*GPNMB, **AHR***
8q	8q21.11 – qter	>37.5%	*PTK2, **RAD21**, MLZE, WISP1, **NOV**, ENPP2, **MYC, PSCA**, PTP4A3, KCNK9, **TPD52**, MMP16, **NBS1**, **FABP5**, E2F5, BAALC, **EBAG9***
12q	12q14.3 – q21.33	25%	***MDM2***
16p	16p12.3 – p13.13	37.5%	*BFAR*
20q	20q11.23 – q13.11	25%	***MMP9*, C20ORF1, *SRC*, *GHRH*, *E2F1*, DNLC2A, BASE, CDC91L1, WFDC2, SLPI, *CYP24*, *BMP7*, *CSE1L***

*HPIN Loss*			
4q	4q34.1	25%	*FAT, MORF4, **CASP3***
6q	6q16.3	37.5%	
8p	8p11.23 – p23.3	>50%	*SFRP1, **NKX3A**, TRIM35, REAM, RB1CC1, **PDGFRL**, FGL1, TNFRSF10B, **LZTS1**, DLC1, MTSG1, **TUSC3**, FLJ32642, MTSS1, **PINX1, DEFB1, CSMD1***
10q	10q23.1	25%	
	10q23.2 – q25.1	25%	***PTEN*, *MXI1*, LGI1, PDCD4, *LAPSER1*, RNF27, SUFU, CASP7, LIMAB1, NEURL**
13q	13q13.1 – q14.13		*RFP2, TSC22, **DBM**, DDX26, **KCNRG***
	13q14.3	25%	*DLEU2, DLEU1, **CHC1L, FAM10A4, FKHR***
16q	16q22.1 – qter	37.5%	*TERF2, **CDH1, DERPC, WWOX**, OKL38, CBFA2T3, **CDH13, WFDC1, MAF, FOXF1, MVD***
18q	18q21.32 – q22.2	25%	*PMAIP1*

*CaP Gain*			
4p	4p13 – p15.1	25%	*UCHL1, CD38*
7p	7p22.1	50%	*
7q	7q11.23	50%	***LIMK1*, CLDN4, *HSPB1***
	7q31.1 – q31.33	25%	*NRCAM, PTPRZ1, POT1*
8q	8q21.11 – qter	>50%	*
16p	16p12.2 – p13.13	25%	*
19p	19p13.11 – pter	25%	*VAV1, RAB3D, ELAVL1, JUN-B, JUN-D,**EPOR**, DRIL1, BSG, ANGPTL4*

*CaP Loss*			
1p	1p22.3	37.5%	
	1p13.1 – p13.2	25%	*ST7L*
5q	5q14.2 – q14.3	25%	
6q	6q16.1	50%	
8p	8p11.22 – p23.3	>50%	*
10q	10q22.2 – q23.33	>37.5%	*UNC5B, BMPR1A, BLNK, **PTEN***
	10q25.3 – q26.11	37.5%	***DMBT1***, TACC2, WDR11, ***FGFR2***, DEC, BCCIP
12p	12p13.2	25%	*CD9*
13q	13q13.3 – q26.11	>50%	BRCA3, KLF5 *
16q	16q24.1 – qter	37.5%	*
18q	18q21.33 – q22.1	25%	*

* indicates the same candidate genes from HPIN. Names in bold indicate those genes that have been implicated in prostate cancer.
