# Supplementary figures and images for: The use of whole genome amplification to study chromosomal changes in prostate cancer: insights into genome-wide signature of preneoplasia associated with cancer progression
Source: BMC Genomics. 2006 Mar 30;7:65. doi: 10.1186/1471-2164-7-65 (PMC1450280; doi:10.1186/1471-2164-7-65)

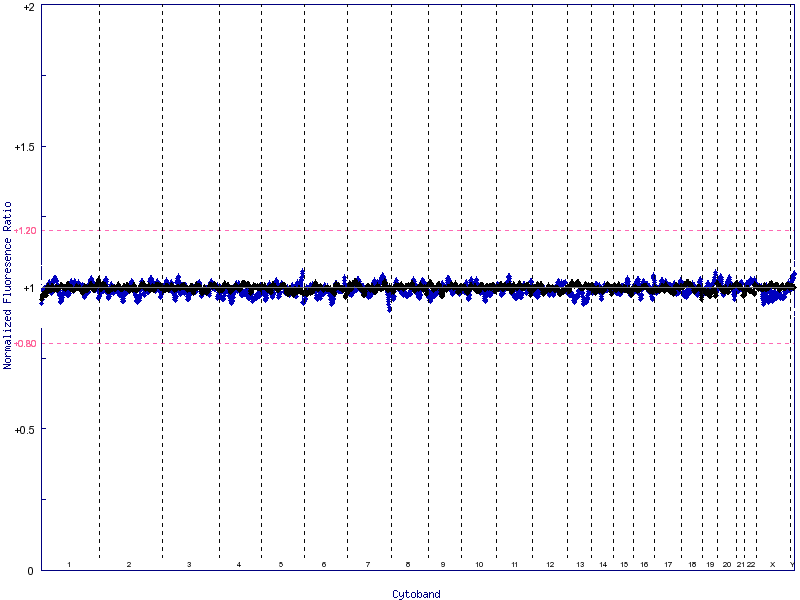

Supplement: Additional File 1 — Genomic comparison of gaCGH results obtained from non-amplified male DNA versus non-amplified male DNA hybridisation and amplified male DNA versus amplified male DNA hybridisation. Blue line indicates the chromosome position plotting of amplified DNA data. Black line indicates the chromosome position plotting of non-amplified DNA data. [file 1471-2164-7-65-S1.png]

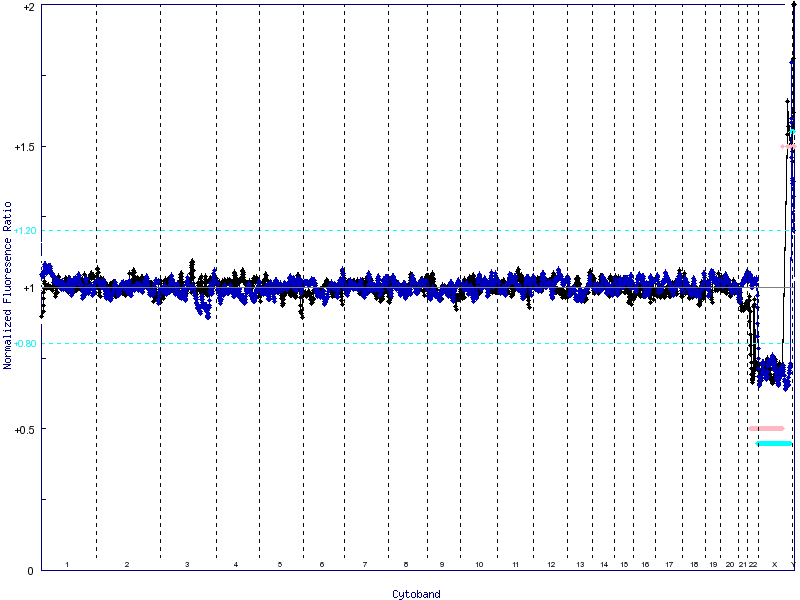

Supplement: Additional File 2 — Genomic comparison of gaCGH results obtained from non-amplified male versus non-amplified female DNA hybridisation and amplified male DNA versus female DNA hybridisation. Blue line indicates the chromosome position plotting of amplified DNA data. Black line indicates the chromosome position plotting of non-amplified DNA data. [file 1471-2164-7-65-S2.png]

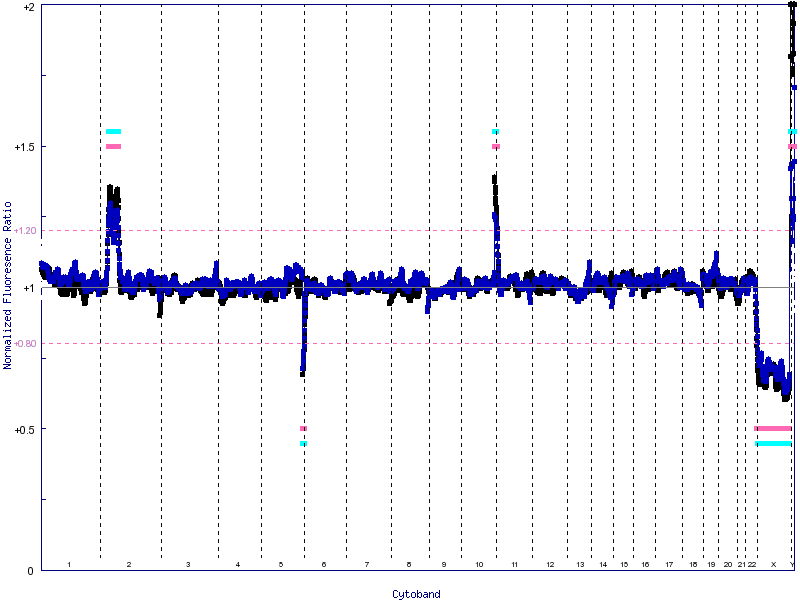

Supplement: Additional File 3 — Genomic comparison of gaCGH results obtained from non-amplified DNA from the colorectal cell line DLD1 versus non-amplified control DNA male hybridisation and amplified DNA from the colorectal cell line DLD1 versus amplified control DNA hybridisation. Blue line indicates the chromosome position plotting of amplified DNA data. Black line indicates the chromosome position plotting of non-amplified DNA data. Detection of gain and loss is shown by cyan and pink bars, corresponding to amplified and non-amplified DNA, respectively. [file 1471-2164-7-65-S3.png]

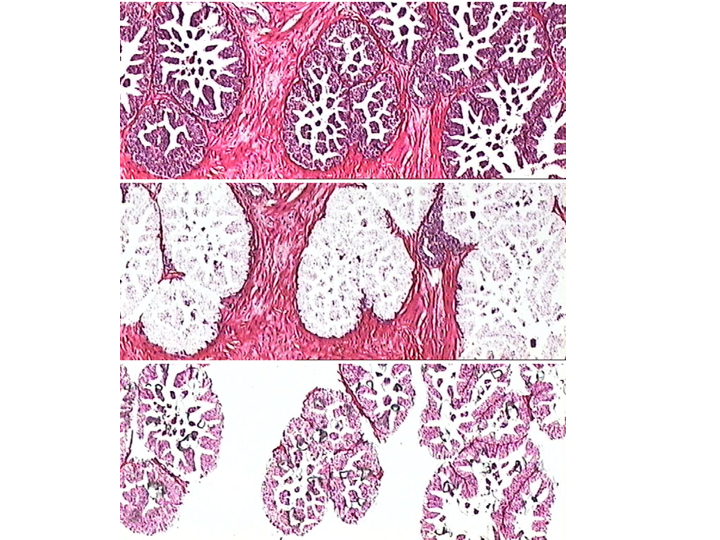

Supplement: Additional File 4 — H&E sections show an example of LCM. Dissection of HPIN. Top picture represents the tissue before dissection, middle picture is after dissection, and bottom picture is the cap tissue. [file 1471-2164-7-65-S4.tiff]
